# Frontiers in chronic fatigue syndrome research: An analysis of the top 100 most influential articles in the field

**DOI:** 10.1097/MD.0000000000035754

**Published:** 2023-11-17

**Authors:** Xingxin Wang, Xuhao Li, Tiantian Dong, Wenyan Yu, Zhixia Jia, Jun Chen

**Affiliations:** a School of Acupuncture-Moxibustion and Tuina, Shandong University of Traditional Chinese Medicine, Jinan, China; b The First Clinical Medical College, Shandong University of Traditional Chinese Medicine, Jinan, China.

**Keywords:** bibliometric analysis, chronic fatigue syndrome, Scimago Graphica, top 100 cited papers, trends

## Abstract

Chronic fatigue syndrome (CFS) is a complex constellation of symptoms that significantly reduces the quality of life among affected individuals and increases public health expenditures. We conducted a search on the Web of Science Core Collection database and selected the top 100 cited articles in the field of CFS. Several literature analysis tools, including CiteSpace 6.1.R6, VOSviewer 1.6.19, and Scimago Graphica 1.0.30, were utilized to integrate the most influential research papers and academic journals in order to obtain a comprehensive understanding of the CFS field. The top 100 highly-cited publications were published in 67 reputable journals, with contributions from 250 institutions across 26 countries/regions involved in CFS research. This demonstrates the extensive attention and coverage of CFS research by high-quality academic journals and institutions, highlighting the interdisciplinary and multidisciplinary nature of CFS studies. The journal with the highest publication volume and total citations was Lancet. The top 5 co-occurring keywords were chronic fatigue syndrome, cognitive behavior therapy, epidemiology, definition, and disorders, indicating the ongoing attention researchers have devoted to the diagnostic criteria and clinical studies of CFS. Cluster analysis results suggested that primary care, infectious retrovirus, gene expression, and metabolomics may become the focal points and trends in future CFS research. The prospective research directions in this field include the search for biological markers, with a particular focus on immunology; the advancement of diagnostic techniques; the screening of risk genes associated with CFS; and the conduct of epidemiological investigations.

## 1. Introduction

Chronic fatigue syndrome (CFS) is a complex constellation of symptoms characterized by long-term physical and mental fatigue.^[1]^ The clinical prevalence of CFS varies greatly (0.8–4.9%), and it significantly reduces the quality of life for affected individuals while also increasing public health expenditures.^[2,3]^ With the advancement of CFS research and the amplification of patient voices, there is a growing concern and attention from the scientific community, medical field, and government agencies worldwide. The underlying mechanisms of CFS are still not fully understood, with various hypotheses including immune system abnormalities, neuroendocrine dysregulation, infections, and psychosocial factors. The treatment goals for CFS focus on symptom relief, functional recovery, and improvement in quality of life, as there is currently no specific curative treatment targeting its root cause. It has been reported that symptomatic drug therapies may alleviate some CFS symptoms, although no therapy has been proven to be consistently effective.^[4,5]^

In 1988, the CDC introduced the concept of CFS into the medical field based on preliminary surveys and expert opinions, providing a clear definition and diagnostic criteria.^[6]^ Since then, CFS has attracted widespread attention and research, gradually becoming an independent disease entity. Despite significant attention over the past 30 years, the etiology and mechanisms of CFS still hold many mysteries. With the outbreak of corona virus disease 2019 (COVID-19), many patients have been experiencing Long COVID, which shares similarities with CFS, as they may undergo prolonged or persistent physical and mental fatigue.^[7]^ Therefore, continuous exploration and research into the complex and enigmatic puzzle of CFS are crucial. Literature metrics have advantages in analyzing and evaluating the research focus, trends, and methodologies in related fields. They can also help researchers identify weak areas of knowledge and provide guidance for future research directions.^[8]^ Unfortunately, we have not identified any high-quality literature metrics analysis of CFS.

We conducted a search in the Web of Science (WOS) Core Collection database to retrieve the top 100 highly cited literature in the CFS field. Various literature analysis tools, such as CiteSpace 6.1.R6, VOSviewer 1.6.19, and Scimago Graphica 1.0.30, were employed to consolidate the most influential research articles and academic journals. This comprehensive approach aimed to acquire a thorough understanding of the CFS domain, encompassing emerging research directions, hot topics, research methodologies, and groundbreaking discoveries. By transforming the data into visual representations, we obtained a panoramic view that offers guidance and inspiration for future studies.

## 2. Materials and methods

### 2.1. Data sources and search strategies

Retrieval of bibliographic data from the WOS Core Collection database was conducted using the following search criteria: publication date between January 1, 2000, and July 1, 2023, and retrieval of the top 100 cited articles (https://www.webofscience.com/wos/woscc/basic-search). The literature search was performed based on the following framework: TI = (Chronic Fatigue Syndromes) OR TI = (Fatigue Syndromes, Chronic) OR TI = (Infectious Mononucleosis-Like Syndrome, Chronic) OR TI = (Infectious Mononucleosis Like Syndrome, Chronic) OR TI = (Royal Free Disease) OR TI = (Chronic Fatigue and Immune Dysfunction Syndrome) OR TI = (Postviral Fatigue Syndrome) OR TI = (Fatigue Syndrome, Postviral) OR TI = (Fatigue Syndromes, Postviral) OR TI = (Postviral Fatigue Syndromes) OR TI = (Syndrome, Postviral Fatigue) OR TI = (Syndromes, Postviral Fatigue) OR TI = (Systemic Exertion Intolerance Disease) OR TI = (Myalgic Encephalomyelitis) OR TI = (Encephalomyelitis, Myalgic) OR TI = (Chronic Fatigue Syndrome) OR TI = (Chronic Fatigue-Fibromyalgia Syndrome) OR TI = (Chronic Fatigue Fibromyalgia Syndrome) OR TI = (Chronic Fatigue-Fibromyalgia Syndromes) OR TI = (Fatigue-Fibromyalgia Syndrome, Chronic) OR TI = (Fatigue-Fibromyalgia Syndromes, Chronic) OR TI = (Chronic Fatigue Disorder) OR TI = (Chronic Fatigue Disorders) OR TI = (Fatigue Disorder, Chronic) OR TI = (Fatigue Disorders, Chronic). The publication types were limited to articles and review articles, and the language was restricted to English.

### 2.2. Inclusion and exclusion criteria

The literature obtained through the retrieval strategy will be sorted in descending order according to the citation frequency. Based on automated and manual checking performed by WXX, LXH, and DTT, duplicate or irrelevant CFS (Chronic Fatigue Syndrome) literature will be excluded. The inclusion of literature in the top 100 citation count will be determined by reading the titles and abstracts. In case of any disagreements in the selection process, CJ will provide assistance for resolution.

### 2.3. Data collection

WXX exported the 29 elements, including Author(s), Title, Source, Times Cited Count, Abstract, Addresses, Affiliations, Document Type, and Keywords, of the included literature using Refworks and Excel formats. The exported data underwent renaming and format conversion to accommodate different software. Spelling errors were manually corrected, and inconsistent descriptions were standardized.

### 2.4. Bibliometric analysis

Utilizing CiteSpace, V.6.2.R4, VOSviewer version 1.6.18, and Scimago Graphica 1.0.35, the potential knowledge information within CFS literature was extracted as data material, and the data was converted into panoramic images. The focus was on bibliometric indicators and knowledge map indicators, including annual publication volume, average citation frequency, total citation frequency, and keyword frequency. Knowledge map indicators encompassed co-occurrence results of authors, institutions, and keywords, as well as clustering, emergence, and timeline diagrams of keywords. The framework and developmental trends of CFS research from 2000 to 2023 were graphically presented, facilitating the analysis of current research hotspots and potential trends in the field of CFS.

## 3. Results

### 3.1. Citation characteristics of the included

#### 3.1.1. Articles.

**Figure 1.**
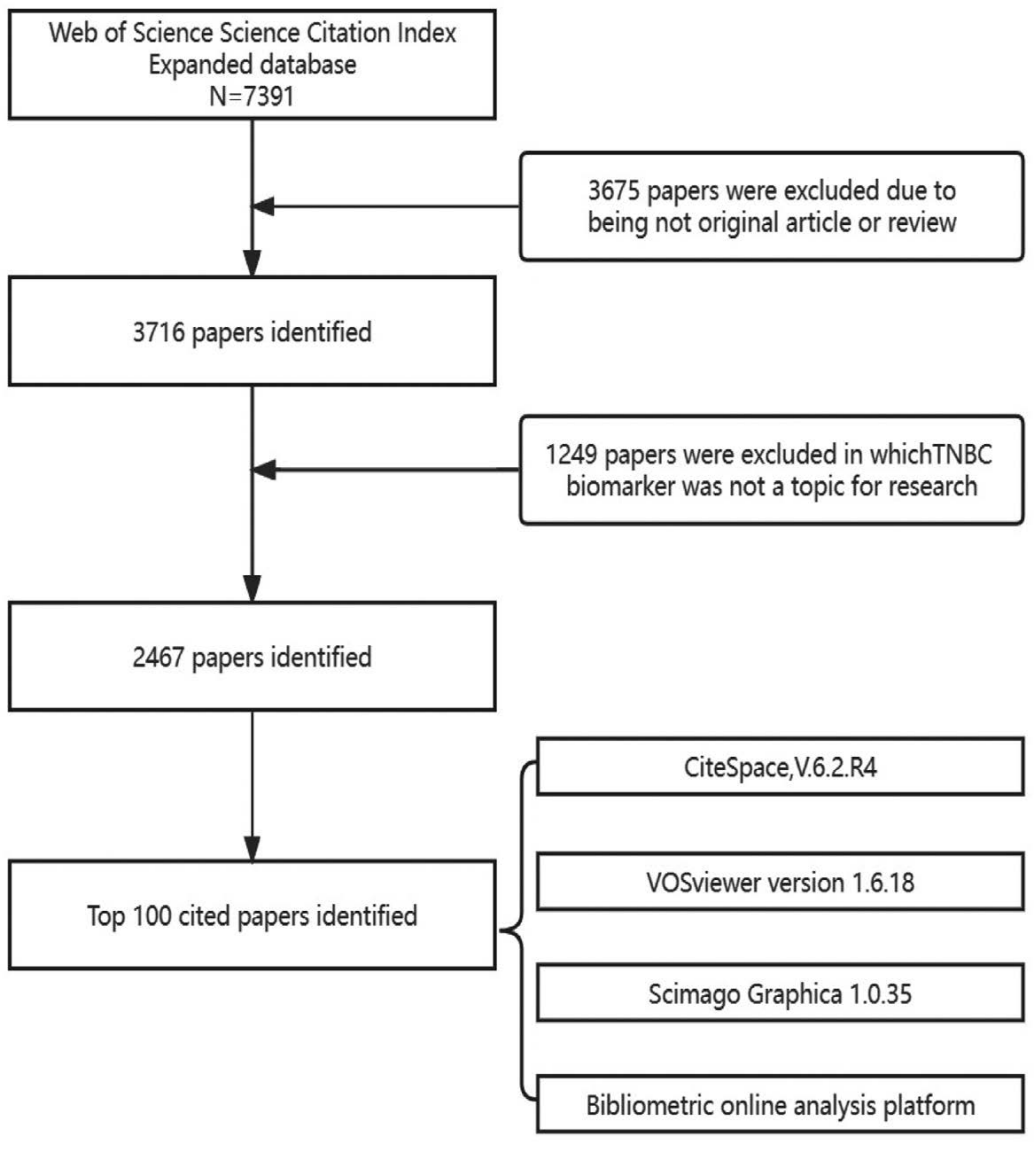
Flowchart of literature screening.

Between 2000 and 2023, a total of 3356 articles on CFS were published. Among them, there were 1053 papers excluding reviews. YWY and JZX reviewed the titles, abstracts, and keywords of the literature, eliminating non-CFS studies, resulting in 1567 relevant research items. These 1567 articles were then sorted in descending order based on their citation frequencies. WXX conducted a final quality check on the literature and ultimately included the top 100 articles that met the criteria for citation frequency. The specific workflow is shown in Figure 1.

Table 1 displays the compilation of the 100 most referenced papers, encompassing a total of 18,818 citations (mean = 188.18). The references amount to a sum of 5918 articles. The paper with the highest citation count is “Myalgic encephalomyelitis: International Consensus Criteria” by Carruthers, BM (662 citations). The International Consensus Criteria establishes the standard for expressing and interpreting the symptoms of CFS, serving as the primary criterion for its identification among clinical physicians, researchers, and other healthcare providers. Subsequently, the following papers have received notable citation attention: “Comparison of adaptive pacing therapy, cognitive behavior therapy, graded exercise therapy, and specialist medical care for chronic fatigue syndrome (PACE): a randomized trial” (582 citations), “A community-based study of chronic fatigue syndrome” (524 citations), and “Chronic fatigue syndrome: A review” (516 citations). Each of the aforementioned articles has been cited more than 500 times.

**Table 1:** The top 100 cited papers in CFS until 2023.

Title	First author	Journal	Total	Year	Average citation
Myalgic encephalomyelitis: International Consensus Criteria	Carruthers, BM	JOURNAL OF INTERNAL MEDICINE	662	2011	55.17
Comparison of adaptive pacing therapy, cognitive behavior therapy, graded exercise therapy, and specialist medical care for chronic fatigue syndrome (PACE): a randomized trial	White, PD	LANCET	582	2011	48.50
A community-based study of chronic fatigue syndrome	Jason, LA	ARCHIVES OF INTERNAL MEDICINE	524	1999	21.83
Chronic fatigue syndrome: A review	Afari, N	AMERICAN JOURNAL OF PSYCHIATRY	516	2003	25.80
Post-infective and chronic fatigue syndromes precipitated by viral and non-viral pathogens: prospective cohort study	Hickie, I	BRITISH MEDICAL JOURNAL	471	2006	27.71
Overlapping conditions among patients with chronic fatigue syndrome, fibromyalgia, and temporomandibular disorder	Aaron, LA	ARCHIVES OF INTERNAL MEDICINE	425	2000	18.48
High rates of autoimmune and endocrine disorders, fibromyalgia, chronic fatigue syndrome and atopic diseases among women with endometriosis: a survey analysis	Sinaii, N	HUMAN REPRODUCTION	418	2002	19.90
Chronic fatigue syndrome	Prins, JB	LANCET	415	2006	24.41
Central sensitization: a biopsychosocial explanation for chronic widespread pain in patients with fibromyalgia and chronic fatigue syndrome	Meeus, M	CLINICAL RHEUMATOLOGY	380	2007	23.75
Cognitive behavior therapy for chronic fatigue syndrome: a multicentre randomized controlled trial	Prins, JB	LANCET	369	2001	16.77
A randomized, double-blind, placebo-controlled pilot study of a probiotic in emotional symptoms of chronic fatigue syndrome	Rao, AV	GUT PATHOGENS	349	2009	24.93
Interventions for the treatment and management of chronic fatigue syndrome - A systematic review	Whiting, P	JAMA-JOURNAL OF THE AMERICAN MEDICAL ASSOCIATION	341	2001	15.50
Identification of ambiguities in the 1994 chronic fatigue syndrome research case definition and recommendations for resolution	Reeves, WC	BMC HEALTH SERVICES RESEARCH	339	2003	16.95
Prevalence and incidence of chronic fatigue syndrome in Wichita, Kansas	Reyes, M	ARCHIVES OF INTERNAL MEDICINE	276	2003	13.80
A systematic review describing the prognosis of chronic fatigue syndrome	Cairns, R	OCCUPATIONAL MEDICINE-OXFORD	241	2005	13.39
Oxidative stress levels are raised in chronic fatigue syndrome and are associated with clinical symptoms	Kennedy, G	FREE RADICAL BIOLOGY AND MEDICINE	241	2005	13.39
The neuroendocrinology of chronic fatigue syndrome	Cleare, AJ	ENDOCRINE REVIEWS	235	2003	11.75
Metabolic features of chronic fatigue syndrome	Naviaux, RK	PROCEEDINGS OF THE NATIONAL ACADEMY OF SCIENCES OF THE UNITED STATES OF AMERICA	228	2016	32.57
Functional neuroimaging correlates of mental fatigue induced by cognition among chronic fatigue syndrome patients and controls	Cook, DB	NEUROIMAGE	218	2007	13.63
Neuroinflammation in Patients with Chronic Fatigue Syndrome/Myalgic Encephalomyelitis: An C-11-(R)-PK11195 PET Study	Nakatomi, Y	JOURNAL OF NUCLEAR MEDICINE	210	2014	23.33
Cognitive behavior therapy for chronic fatigue syndrome in adults	Price, JR	COCHRANE DATABASE OF SYSTEMATIC REVIEWS	203	2008	13.53
Immunological aspects of chronic fatigue syndrome	Lorusso, L	AUTOIMMUNITY REVIEWS	203	2009	14.50
Reduced diversity and altered composition of the gut microbiome in individuals with myalgic encephalomyelitis/chronic fatigue syndrome	Giloteaux, L	MICROBIOME	202	2016	28.86
Salivary cortisol response to awakening in chronic fatigue syndrome	Roberts, ADL	BRITISH JOURNAL OF PSYCHIATRY	196	2004	10.32
Cytokine signature associated with disease severity in chronic fatigue syndrome patients	Montoya, JG	PROCEEDINGS OF THE NATIONAL ACADEMY OF SCIENCES OF THE UNITED STATES OF AMERICA	195	2017	32.50
Chronic Fatigue Syndrome - A clinically empirical approach to its definition and study	Reeves, WC	BMC MEDICINE	188	2005	10.44
Childhood Trauma and Risk for Chronic Fatigue Syndrome Association With Neuroendocrine Dysfunction	Heim, C	ARCHIVES OF GENERAL PSYCHIATRY	185	2009	13.21
Chronic fatigue syndrome and mitochondrial dysfunction	Myhill, S	INTERNATIONAL JOURNAL OF CLINICAL AND EXPERIMENTAL MEDICINE	183	2009	13.07
Failure to Detect the Novel Retrovirus XMRV in Chronic Fatigue Syndrome	Erlwein, O	PLOS ONE	178	2010	13.69
Pain Physiology Education Improves Pain Beliefs in Patients With Chronic Fatigue Syndrome Compared With Pacing and Self-Management Education: A Double-Blind Randomized Controlled Trial	Meeus, M	ARCHIVES OF PHYSICAL MEDICINE AND REHABILITATION	171	2010	13.15
Nitrosative Stress, Hypernitrosylation, and Autoimmune Responses to Nitrosylated Proteins: New Pathways in Neuroprogressive Disorders Including Depression and Chronic Fatigue Syndrome	Morris, G	MOLECULAR NEUROBIOLOGY	171	2017	28.50
Hypothalamic-pituitary-adrenal axis dysfunction in chronic fatigue syndrome	Papadopoulos, AS	NATURE REVIEWS ENDOCRINOLOGY	169	2012	15.36
Immunological abnormalities as potential biomarkers in Chronic Fatigue Syndrome/Myalgic Encephalomyelitis	Brenu, EW	JOURNAL OF TRANSLATIONAL MEDICINE	167	2011	13.92
Interstitial Cystitis/Painful Bladder Syndrome and Associated Medical Conditions With an Emphasis on Irritable Bowel Syndrome, Fibromyalgia and Chronic Fatigue Syndrome	Nickel, JC	JOURNAL OF UROLOGY	165	2010	12.69
Basal circadian and pulsatile ACTH and cortisol secretion in patients with fibromyalgia and/or chronic fatigue syndrome	Crofford, LJ	BRAIN BEHAVIOR AND IMMUNITY	165	2004	8.68
Early adverse experience and risk for chronic fatigue syndrome - Results from a population-based study	Heim, C	ARCHIVES OF GENERAL PSYCHIATRY	165	2006	9.71
Case definitions for chronic fatigue syndrome/myalgic encephalomyelitis (CFS/ME): a systematic review	Brurberg, KG	BMJ OPEN	163	2014	18.11
Cognitive behavior therapy for adolescents with chronic fatigue syndrome: randomized controlled trial	Stulemeijer, M	BMJ-BRITISH MEDICAL JOURNAL	162	2005	9.00
Low-dose hydrocortisone in chronic fatigue syndrome: a randomized crossover trial	Cleare, AJ	LANCET	161	1999	6.71
Absence of xenotropic murine leukaemia virus-related virus in UK patients with chronic fatigue syndrome	Groom, HCT	RETROVIROLOGY	159	2010	12.23
Increased serum IgA and IgM against LPS of enterobacteria in chronic fatigue syndrome (CFS): Indication for the involvement of gram-negative enterobacteria in the etiology of CFS and for the presence of an increased gut-intestinal permeability	Maes, M	JOURNAL OF AFFECTIVE DISORDERS	159	2007	9.94
Ambulatory monitoring of physical activity and symptoms in fibromyalgia and chronic fatigue syndrome	Kop, WJ	ARTHRITIS AND RHEUMATISM	157	2005	8.72
Identifying physical activity patterns in chronic fatigue syndrome using actigraphic assessment	van der Werf, SP	JOURNAL OF PSYCHOSOMATIC RESEARCH	156	2000	6.78
Increase in prefrontal cortical volume following cognitive behavioral therapy in patients with chronic fatigue syndrome	de Lange, FP	BRAIN	156	2008	10.40
Autonomic nervous system dysfunction in adolescents with postural orthostatic tachycardia syndrome and chronic fatigue syndrome is characterized by attenuated vagal baroreflex and potentiated sympathetic vasomotion	Stewart, JM	PEDIATRIC RESEARCH	154	2000	6.70
Plasma cytokines in women with chronic fatigue syndrome	Fletcher, MA	JOURNAL OF TRANSLATIONAL MEDICINE	152	2009	10.86
Sympathetic Nervous System Dysfunction in Fibromyalgia, Chronic Fatigue Syndrome, Irritable Bowel Syndrome, and Interstitial Cystitis A Review of Case-Control Studies	Martinez-Martinez, LA	JCR-JOURNAL OF CLINICAL RHEUMATOLOGY	151	2014	16.78
A formal analysis of cytokine networks in Chronic Fatigue Syndrome	Broderick, G	BRAIN BEHAVIOR AND IMMUNITY	150	2010	11.54
Mechanisms underlying fatigue: a voxel-based morphometric study of chronic fatigue syndrome	Okada, T	BMC NEUROLOGY	149	2004	7.84
Chronic fatigue syndrome: assessment of increased oxidative stress and altered muscle excitability in response to incremental exercise	Jammes, Y	JOURNAL OF INTERNAL MEDICINE	149	2005	8.28
REDUCED PRESSURE PAIN THRESHOLDS IN RESPONSE TO EXERCISE IN CHRONIC FATIGUE SYNDROME BUT NOT IN CHRONIC LOW BACK PAIN AN EXPERIMENTAL STUDY	Meeus, M	JOURNAL OF REHABILITATION MEDICINE	146	2010	11.23
Prevalence of myalgic encephalomyelitis/chronic fatigue syndrome (ME/CFS) in three regions of England: a repeated cross-sectional study in primary care	Nacul, LC	BMC MEDICINE	146	2011	12.17
Incidence, prognosis, and risk factors for fatigue and chronic fatigue syndrome in adolescents: A prospective community study	Rimes, KA	PEDIATRICS	146	2007	9.13
Chronic fatigue syndrome, the immune system and viral infection	Bansal, AS	BRAIN BEHAVIOR AND IMMUNITY	146	2012	13.27
The neuroendocrinology of chronic fatigue syndrome and fibromyalgia	Parker, AJR	PSYCHOLOGICAL MEDICINE	145	2001	6.59
Interventions for the treatment, management and rehabilitation of patients with chronic fatigue syndrome/myalgic encephalomyelitis: an updated systematic review	Chambers, D	JOURNAL OF THE ROYAL SOCIETY OF MEDICINE	144	2006	8.47
Orthostatic intolerance and chronic fatigue syndrome associated with Ehlers-Danlos syndrome	Rowe, PC	JOURNAL OF PEDIATRICS	143	1999	5.96
Symptoms of autonomic dysfunction in chronic fatigue syndrome	Newton, JL	QJM-AN INTERNATIONAL JOURNAL OF MEDICINE	140	2007	8.75
Effectiveness of internet-based cognitive behavioral treatment for adolescents with chronic fatigue syndrome (FITNET): a randomized controlled trial	Nijhof, SL	LANCET	139	2012	12.64
Randomised controlled trial of patient education to encourage graded exercise in chronic fatigue syndrome	Powell, P	BMJ-BRITISH MEDICAL JOURNAL	136	2001	6.18
Integrated Weighted Gene Co-expression Network Analysis with an Application to Chronic Fatigue Syndrome	Presson, AP	BMC SYSTEMS BIOLOGY	135	2009	9.64
High-throughput 16S rRNA gene sequencing reveals alterations of intestinal microbiota in myalgic encephalomyelitis/chronic fatigue syndrome patients	Fremont, M	ANAEROBE	135	2013	13.50
Specific oxidative alterations in vastus lateralis muscle of patients with the diagnosis of chronic fatigue syndrome	Fulle, S	FREE RADICAL BIOLOGY AND MEDICINE	134	2000	5.83
Myalgic Encephalomyelitis/Chronic Fatigue Syndrome - Evidence for an autoimmune disease	Sotzny, F	AUTOIMMUNITY REVIEWS	133	2018	26.60
Antibodies to beta adrenergic and muscarinic cholinergic receptors in patients with Chronic Fatigue Syndrome	Loebel, M	BRAIN BEHAVIOR AND IMMUNITY	133	2016	19.00
Benefit from B-Lymphocyte Depletion Using the Anti-CD20 Antibody Rituximab in Chronic Fatigue Syndrome. A Double-Blind and Placebo-Controlled Study	Fluge, O	PLOS ONE	133	2011	11.08
Fatigue and chronic fatigue syndrome-like complaints in the general population	van’t Leven, M	EUROPEAN JOURNAL OF PUBLIC HEALTH	132	2010	10.15
Frequent HHV-6 reactivation in multiple sclerosis (MS) and chronic fatigue syndrome (CFS) patients	Ablashi, DV	JOURNAL OF CLINICAL VIROLOGY	132	2000	5.74
A rating scale for fibromyalgia and chronic fatigue syndrome (the FibroFatigue scale)	Zachrisson, O	JOURNAL OF PSYCHOSOMATIC RESEARCH	129	2002	6.14
Increased 8-hydroxy-deoxyguanosine, a marker of oxidative damage to DNA, in major depression and myalgic encephalomyelitis chronic fatigue syndrome	Maes, M	NEUROENDOCRINOLOGY LETTERS	128	2009	9.14
Absence of evidence of Xenotropic Murine Leukemia Virus-related virus infection in persons with Chronic Fatigue Syndrome and healthy controls in the United States	Switzer, WM	RETROVIROLOGY	127	2010	9.77
Systematic review and meta-analysis of the prevalence of chronic fatigue syndrome/myalgic encephalomyelitis (CFS/ME)	Lim, EJ	JOURNAL OF TRANSLATIONAL MEDICINE	127	2020	42.33
Victimization in chronic fatigue syndrome and fibromyalgia in tertiary care - A controlled study on prevalence and characteristics	Van Houdenhove, B	PSYCHOSOMATICS	126	2001	5.73
In the mind or in the brain? Scientific evidence for central sensitization in chronic fatigue syndrome	Nijs, J	EUROPEAN JOURNAL OF CLINICAL INVESTIGATION	126	2012	11.45
Chronic fatigue syndrome	Devanur, LD	JOURNAL OF CLINICAL VIROLOGY	126	2006	7.41
Randomised controlled trial of graded exercise in chronic fatigue syndrome	Wallman, KE	MEDICAL JOURNAL OF AUSTRALIA	123	2004	6.47
Heart rate variability in patients with fibromyalgia and patients with chronic fatigue syndrome: A systematic review	Meeus, M	SEMINARS IN ARTHRITIS AND RHEUMATISM	123	2013	12.30
Increased ventricular lactate in chronic fatigue syndrome. III. Relationships to cortical glutathione and clinical symptoms implicate oxidative stress in disorder pathophysiology	Shungu, DC	NMR IN BIOMEDICINE	122	2012	11.09
Objective evidence of cognitive complaints in Chronic Fatigue Syndrome: A BOLD fMRI study of verbal working memory	Lange, G	NEUROIMAGE	121	2005	6.72
Premorbid overactive lifestyle in chronic fatigue syndrome and fibromyalgia - An etiological factor or proof of good citizenship?	Van Hudenhove, B	JOURNAL OF PSYCHOSOMATIC RESEARCH	121	2001	5.50
Adolescent Chronic Fatigue Syndrome: Prevalence, Incidence, and Morbidity	Nijhof, SL	PEDIATRICS	121	2011	10.08
Chronic Fatigue Syndrome After Infectious Mononucleosis in Adolescents	Katz, BZ	PEDIATRICS	121	2009	8.64
Increased D-Lactic Acid Intestinal Bacteria in Patients with Chronic Fatigue Syndrome	Sheedy, JR	IN VIVO	121	2009	8.64
Gray matter volume reduction in the chronic fatigue syndrome	de Lange, FP	NEUROIMAGE	120	2005	6.67
Diffuse noxious inhibitory control is delayed in chronic fatigue syndrome: An experimental study	Meeus, M	PAIN	120	2008	8.00
Relationship between musculoskeletal symptoms and blood markers of oxidative stress in patients with chronic fatigue syndrome	Vecchiet, J	NEUROSCIENCE LETTERS	119	2003	5.95
Moderate Exercise Increases Expression for Sensory, Adrenergic, and Immune Genes in Chronic Fatigue Syndrome Patients But Not in Normal Subjects	Light, AR	JOURNAL OF PAIN	119	2009	8.50
Long COVID and Myalgic Encephalomyelitis/Chronic Fatigue Syndrome (ME/CFS)-A Systemic Review and Comparison of Clinical Presentation and Symptomatology	Wong, TL	MEDICINA-LITHUANIA	118	2021	59.00
Chronic viral infections in myalgic encephalomyelitis/chronic fatigue syndrome (ME/CFS)	Rasa, S	JOURNAL OF TRANSLATIONAL MEDICINE	118	2018	23.60
Strength and physiological response to exercise in patients with chronic fatigue syndrome	Fulcher, KY	JOURNAL OF NEUROLOGY NEUROSURGERY AND PSYCHIATRY	114	2000	4.96
Neuroendocrine perturbations in fibromyalgia and chronic fatigue syndrome	Neeck, G	RHEUMATIC DISEASE CLINICS OF NORTH AMERICA	113	2000	4.91
Is a full recovery possible after cognitive behavioral therapy for chronic fatigue syndrome?	Knoop, H	PSYCHOTHERAPY AND PSYCHOSOMATICS	113	2007	7.06
Fear of movement and avoidance behavior toward physical activity in chronic-fatigue syndrome and fibromyalgia: state of the art and implications for clinical practice	Nijs, J	CLINICAL RHEUMATOLOGY	112	2013	11.20
Protocol for the PACE trial: A randomized controlled trial of adaptive pacing, cognitive behavior therapy, and graded exercise as supplements to standardized specialist medical care versus standardized specialist medical care alone for patients with the chronic fatigue syndrome/myalgic encephalomyelitis or encephalopathy	White, PD	BMC NEUROLOGY	112	2007	7.00
Prevalence of xenotropic murine leukaemia virus-related virus in patients with chronic fatigue syndrome in the Netherlands: retrospective analysis of samples from an established cohort	van Kuppeveld, FJM	BMJ-BRITISH MEDICAL JOURNAL	111	2010	8.54
Metabolic profiling indicates impaired pyruvate dehydrogenase function in myalgic encephalopathy/chronic fatigue syndrome	Fluge, O	JCI INSIGHT	111	2016	15.86
Orthostatic intolerance in adolescent chronic fatigue syndrome	Stewart, JM	PEDIATRICS	111	1999	4.63
Gene expression in peripheral blood mononuclear cells from patients with chronic fatigue syndrome	Kaushik, N	JOURNAL OF CLINICAL PATHOLOGY	109	2005	6.06
Exercise capacity in chronic fatigue syndrome	De Becker, P	ARCHIVES OF INTERNAL MEDICINE	108	2000	4.70
Stress-induced changes in LPS-induced pro-inflammatory cytokine production in chronic fatigue syndrome	Gaab, J	PSYCHONEUROENDOCRINOLOGY	106	2005	5.89

#### 3.1.2. Year of publication and citation.

**Figure 2.**
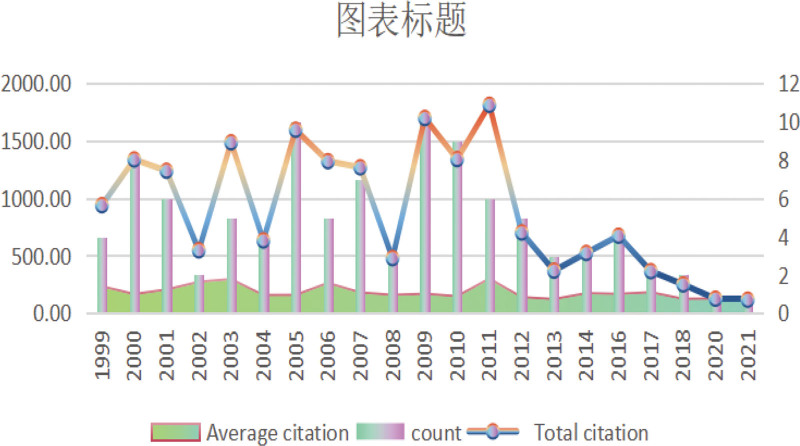
Year of publication and citation.

In order to portray the developmental trajectory of CFS, we have constructed a diagram (Fig. 2) based on the annual publication volume, average citation frequency, and total citation count of the included literature. These publications are primarily concentrated between 2006 and 2016, during which a substantial number of high-quality CFS research achievements were generated, attracting widespread international attention. However, this ranking did not consider the papers from 2022 and 2023. This temporal limitation represents one of the common issues affecting current visualization analyses. At the conclusion of the article, we address this limitation and provide relevant insights. The year 2011 demonstrated the highest total citation count and average citation frequency, indicating the vital significance of these studies in advancing the scientific understanding of CFS, encompassing epidemiological investigations, diagnostic criteria, treatment options, potential biomarkers, and the unique challenges faced by adolescent patients.^[1,9–13]^ The years with the highest publication volume were 2005 and 2009 (N = 10), which may be attributed to increased research funding, a growing interest among researchers in this field, and the occurrence of significant events.

### 3.2. Distribution per journal

**Figure 3.**
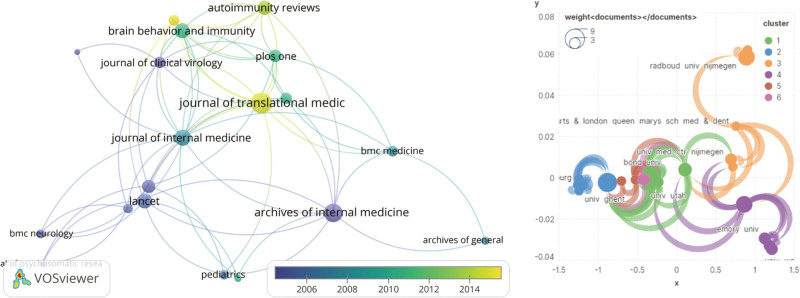
Network visualization of the Journal that contributed to the top 100 cited papers.

In the top 100 highly cited publications, which were published in 67 prestigious journals, it is demonstrated that various academic journals have shown attention and coverage towards CFS research, highlighting its interdisciplinary and multidisciplinary nature. The journal with the highest number of publications is Lancet (N = 5), focusing on clinical randomized controlled trials aimed at providing empirical evidence for the management and treatment of CFS. Following Lancet are Archives of Internal Medicine, Brain Behavior and Immunity, Journal of Translational Medicine, and Pediatrics (N = 4). Lancet also has the highest total citation frequency (N = 1666). According to data statistics, The American Journal of Psychiatry has the highest average citation frequency (1 paper, 516 citations). This journal published a comprehensive review article titled “Chronic fatigue syndrome: A review,” covering various aspects of CFS such as symptoms, diagnosis, etiology, and management approaches. The review highlights that CFS is unlikely to be caused or sustained by a single medication, thus necessitating a comprehensive multidisciplinary approach for its management. This perspective provides reference and inspiration for introducing alternative therapeutic approaches. Figure 3 presents the top 10 prolific journals in the field of CFS, which have published a significant number of papers and are widely cited within the CFS research community. By analyzing the citation relationships, we can gain insights into the academic interactions and knowledge dissemination among different journals, further promoting progress and collaboration in the field of CFS research.

**Figure 4.**
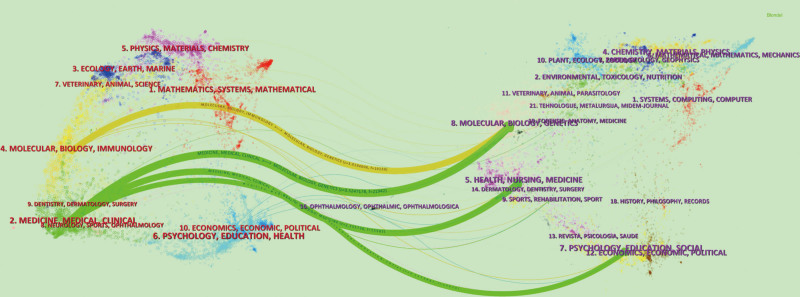
CiteSpace-based dual map overlay of journals connected to the CFS field. CFS = chronic fatigue syndrome.

To further investigate the patterns of academic interaction and knowledge dissemination among different journals, we created a double-journal overlay map (Fig. 4). In this figure, we can observe 4 major reference paths: MOLECULAR BIOLOGY/IMMUNOLOGY - MOLECULAR BIOLOGY/GENETICS; MEDICINE/MEDICAL/CLINICAL and MOLECULAR BIOLOGY/GENETICS; MEDICINE/MEDICAL/CLINICAL→HEALTH/NURSING/MEDICINE; MEDICINE/MEDICAL/CLINICAL - tended to/EDUCATION/SOCIAL. These relationships are organized based on their Z-relationships and are represented by different path colors (Table 2). It is evident from the map that CFS frontier literature is predominantly published in the field of MEDICINE/MEDICAL/CLINICAL, with the main references originating from MOLECULAR BIOLOGY/GENETICS. This information can serve as a valuable resource for conducting high-quality literature searches and exploring new research directions in the field of CFS.

**Table 2:** The Z distribution of CiteSpace-based dual map overlay.

Citing region	Cited region	*Z* score
MEDICINE, MEDICAL, CLINICAL	MOLECULAR/BIOLOGY/GENETICS	3.525
MOLECULAR,BIOLOGY,IMMUNOLOGY	MOLECULAR/BIOLOGY/GENETICS	3.019
MEDICINE, MEDICAL, CLINICAL	HEALTH, NURSING, MEDICINE	2.336
MEDICINE, MEDICAL, CLINICAL	PSYCHOLOGY, EDUCATION,SOCIAL	2.157

### 3.3. Author and coauthor analysis

**Figure 5.**
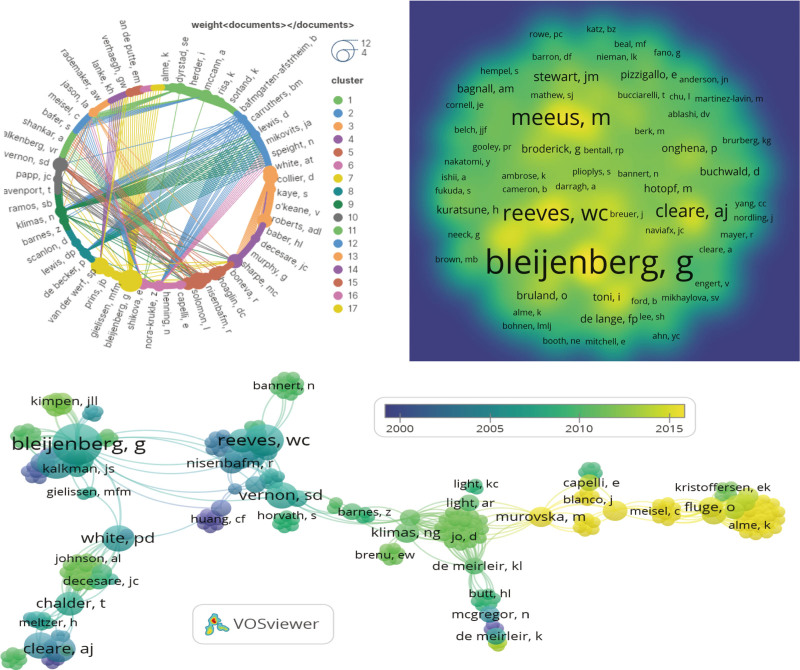
Network visualization of authors that contributed to the top 100 cited papers.

There was active cooperation among clusters of 10 distinct hues in this study (Fig. 5). A total of 555 authors participated in the study. To avoid any uncertainty with author abbreviations, we modified the author’s name format. For example, “Davenport, T” was changed to “Davenport, Trace.” Bleijenberg, G had the largest number of papers (N = 12), followed by Reeves, WC (N = 7), van der Meer, JWM (N = 7), and Meeus, M (N = 7). The network centered around Bleijenberg, G had a total connection strength of 64, and the largest author group consisted of 47 authors. The most frequently cited authors were Bleijenberg, G (N = 2302), followed by Reeves, WC (N = 1720) and van der Meer, JWM (N = 1419). The average number of citations per paper for the 21 authors was the highest (1 paper, 662.00 citations). In the field of CFS research, core figures like Bleijenberg, G, Meeus, M, and Reeves, WC have had a significant influence and have played leading and exemplary roles. Additionally, emerging scholars such as Murovska, M., Fluge, Oystein, and Mella, Olav have seen a significant increase in their citation frequency in recent years. Collectively, this group of scholars has shown a strong interest in understanding the diagnostic criteria, underlying autoimmune components, treatment methods, viral infections, and metabolic dysfunction associated with CFS.

### 3.4. Distribution of countries/regions and institutions

#### 3.4.1. Countries/regions.

A total of 250 institutions participated in the study on CFS, representing 26 countries.^[14–16]^ The United States (N = 37) has published the highest number of papers, making significant contributions to the understanding of diagnostic criteria and the pathogenesis of CFS. This country also plays a pivotal role in driving CFS research forward and enhancing the quality of life for CFS patients worldwide. The United Kingdom (N = 32) and Belgium (N = 27) rank second and third respectively in terms of the number of published papers. Among the 26 countries/regions involved, the United States received the highest number of citations (N = 8004), followed by the United Kingdom (N = 5672) and Belgium (N = 2656). These countries/regions formed 3 main clustering areas. The United States has established the largest national cooperation network, encompassing 23 countries. It exhibits a Total link strength of 958 and a Centrality value of 0.30, indicating strong collaboration and significant influence in this field. The United Kingdom (N = 23) and Belgium (N = 23) subsequently follow suit.

**Figure 6.**
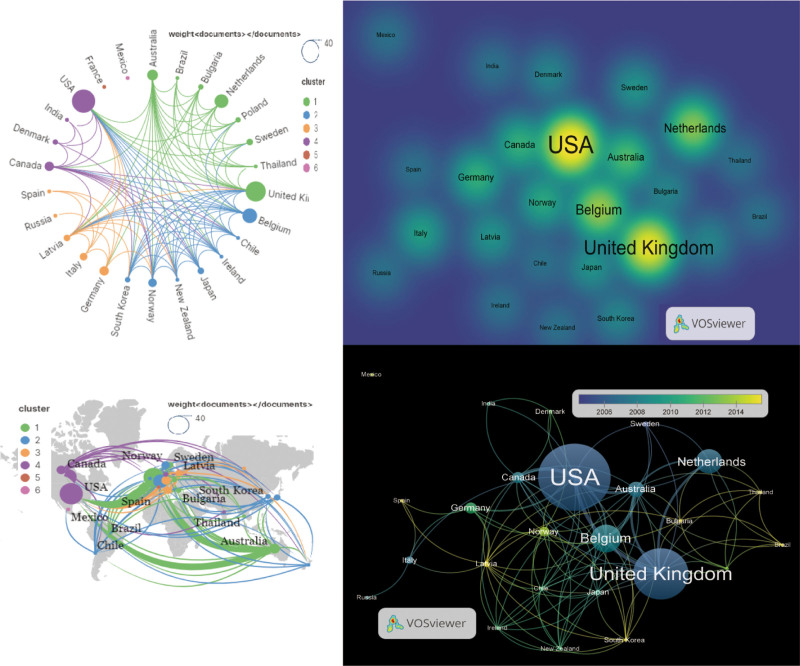
Networks showing the collaboration among Countries.

Figure 6 illustrates a visual representation of the country map, demonstrating the mutual relationships and the level of cooperation between different countries or regions. The thickness of the lines corresponds to the degree of cooperation. In recent years, 6 new countries have joined the CFS study, accounting for 23% of the total number of countries involved. This observation reflects the considerable attention and rapid development of CFS on an international scale. It is anticipated that more countries, notably South Korea, Spain, and Latvia, will publish impactful findings and contribute significantly to the progress of CFS research. Over time, it is expected that an increasing number of countries will actively engage in and further advance CFS research.

#### 3.4.2. Institutions.

**Figure 7.**
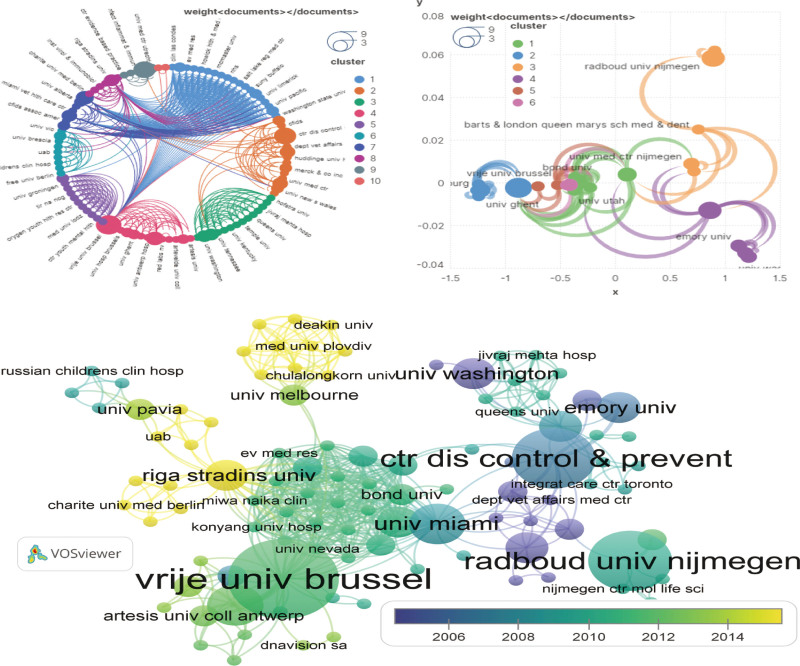
Network visualization of the institutions that contributed to the top 100 cited papers.

In terms of the total number of publications, Vrije Univ Brussel exhibits the highest publication count (N = 8), with a primary research focus on clinical trials and proposing the correlation between central sensitization and CFS. Subsequently, Inst Psychiat (N = 7), Radboud Univ Nijmegen (N = 6), Kings Coll London (N = 6), and Ctr Dis Control & Prevent (N = 6) follow suit. Vrije Univ Brussel garners the highest frequency of citations (N = 1550), trailed by Ctr Dis Control & Prevent (N = 1424), and Kings Coll London (N = 1343). The largest collaborative network of institutions encompasses Ctr Dis Control & Prevent, encompassing 144 institutions, succeeded by Univ Miami and Riga Stradins Univ with 141 and 132 institutions correspondingly. The 250 institutions were collectively organized into 8 main clusters (Fig. 7). Notably, the centrality of Ctr Dis Control & Prevent is 0.30, significantly surpassing other institutions, denoting its robust linkages and substantial influence in this field. Univ Bergen, Riga Stradins Univ, and HAFkeland Hosp emerge as the foremost institutions in terms of recent paper publications, with their research emphasizing metabolomics analysis, chronic viral infections, autoimmunity, and pathogenesis. This correspondence with keyword analysis suggests that forthcoming research will continue to concentrate in these domains.

### 3.5. Research direction

According to the WoS classification, the included literature was categorized into 34 research topics (Table 3). Among these topics, “Medicine, General & Internal” (N = 24) emerged as the most significant research direction, which aligns with the findings depicted in Figure 4. Furthermore, both “Medicine, Research & Experimental” (N = 7) and “Psychiatry” (N = 7) are also regarded as significant research domains. These data demonstrate the focal areas of interest in the research and the relative importance of each topic. For a more comprehensive understanding, please refer to Table 3 for detailed data information.

**Table 3:** WOS categories in the top 100 cited papers on CFS.

WoS categories	Number
Medicine, General & Internal	24
Medicine, Research & Experimental	7
Psychiatry	7
Pediatrics	6
Rheumatology	6
Immunology; Neurosciences; Psychiatry	4
Multidisciplinary Sciences	4
Virology	4
Neurosciences; Neuroimaging; Radiology, Nuclear Medicine & Medical Imaging	3

CFS = chronic fatigue syndrome.

### 3.6. Keywords co-occurrence, clusters and bursts

**Figure 8.**
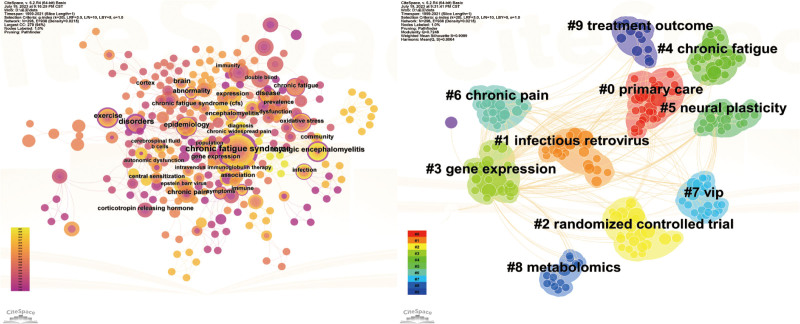
Network visualization of the co-occurring keywords and clustering analysis that contributed to the top 100 cited papers.

Using CiteSpace, V.6.2.R4, the selection criteria were adjusted to *K* = 20, resulting in a total of 296 keywords (Table 4, Fig. 8). The most common terms identified were CFS (N = 31), cognitive behavior therapy (N = 9), epidemiology (N = 8), definition (N = 8), disorders (N = 7), chronic fatigue (N = 7), and double blind (N = 7). This highlights the significance of diagnostic criteria and clinical research in the study of CFS. The most prevalent clinical manifestations include chronic fatigue (N = 7) and chronic pain (N = 6). Currently, the etiology and mechanisms of CFS remain unclear; however, scholars have attempted to investigate the mechanisms of CFS from various perspectives such as immune (gene expression, N = 5), endocrine (pituitary adrenal axis, N = 5), and oxidative stress (N = 6). Due to the complexity of CFS, there is currently no specific medication, and complementary and alternative therapies play a significant role, such as cognitive behavior therapy (N = 9) and exercise (N = 6). In terms of specific diseases, the most commonly studied ones are CFS (N = 31), myalgic encephalomyelitis (N = 6), infection (N = 5), fibromyalgia (N = 5), and multiple sclerosis (N = 4).

**Table 4:** Top 15 co-occurring keywords in the top 100 cited papers on CFS.

Rank	Keyword	Occurrences
1	chronic fatigue syndrome	31
2	cognitive behavior therapy	9
3	definition	8
4	epidemiology	8
5	double blind	7
6	disorders	7
7	chronic fatigue	7
8	epstein barr virus	6
9	chronic pain	6
10	expression	6
11	exercise	6
12	myalgic encephalomyelitis	6
13	oxidative stress	6
14	prevalence	6
15	low dose hydrocortisone	6

CFS = chronic fatigue syndrome.

The focus areas and research directions within the field of CFS can be clearly identified through keyword clustering in the literature (Fig. 8). The prevalence, incidence, and morbidity of CFS are associated with the largest cluster indicated by the color red. The orange cluster primarily investigates the risk and mechanisms of CFS development due to viral infections. The yellow cluster mainly includes double-blind randomized controlled trials related to clinical research on CFS. The green cluster encompasses gene expression, diagnosis, CFS, myalgic encephalomyelitis, and neutrophils. All keywords can be categorized into 22 clusters, with the first ten clusters as follows: cluster 0 (primary care), cluster 1 (infectious retrovirus), cluster 2 (randomized controlled trial), cluster 3 (gene expression), cluster 4 (chronic fatigue), cluster 5 (neural plasticity), cluster 6 (chronic pain), cluster 7 (vip), cluster 8 (metabolomics), and cluster 9 (treatment outcome).

**Figure 9.**
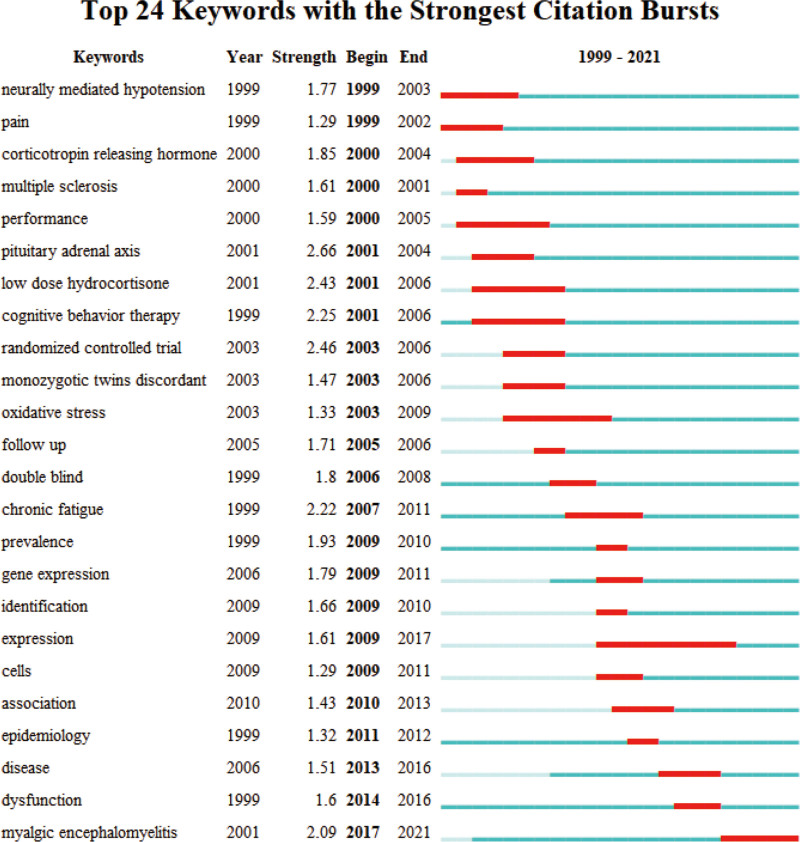
Keyword clustering analysis of the top 100 cited papers changes by year.

**Figure 10.**
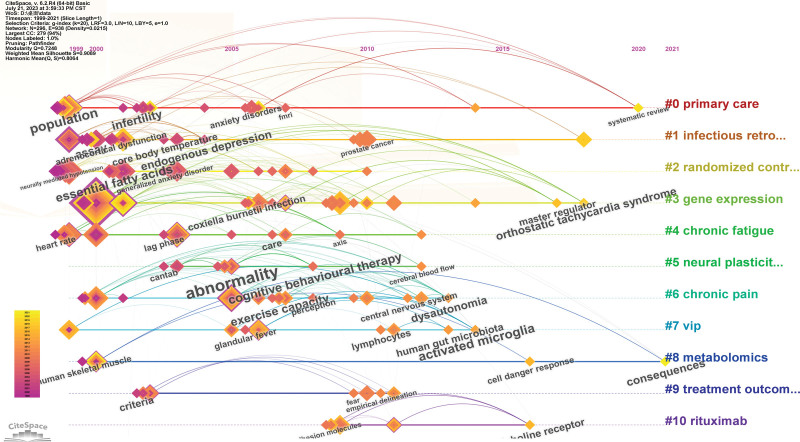
Network visualization of the keywords with the strongest citation.

We employed CFS keyword mapping to facilitate the identification of trends and modifications in keywords, allowing for an analysis of the overall development trajectory (Figs. 9 and 10, Table 5). Epidemiological investigation has consistently remained a research focal point within the domain of CFS. In recent years, attention has also been directed towards the healthcare management of CFS patients, the correlation between long-haul COVID-19 and CFS, as well as the establishment of international standards for CFS. The integration of big data retrieval and retrospective literature analysis has emerged as a novel research avenue, with scholars aiming to identify new areas of investigation through this approach. Cluster analysis results suggest that primary care, infectious retrovirus, gene expression, and metabolomics may represent the upcoming focus and trends in CFS research, aligning with our analysis of publications from emerging institutions over the course of several years.

**Table 5:** Clusters of references cooccurring in the top 100 cited papers on CFS.

Cluster	Size	Year	LLR
0	30	2006	Primary care; impact; endometriosis; community; endocrine
1	27	2004	Infectious retrovirus; corticotropin releasing hormone; xmrv; hypothalamic pituitary; prostate cancer
2	26	2002	Randomized controlled trial; double blind; cognitive behavior therapy; graded exercise; placebo controlled trial
3	26	2010	Gene expression; diagnosis; chronic fatigue syndrome; myalgic encephalomyelitis; neutrophils
4	26	2005	Chronic fatigue; oxidative stress; muscle pain thresholds; neurodegenerationabuse
5	25	2006	Neural plasticityvolumetric mrivoxel-based morphometry; gray matter increase; cognitive behavioral therapy
6	25	2007	Chronic pain; fibromyalgia; pain; spatial summation; cortisol
7	22	2010	Vip; t cells; myalgic encephalomyelitis (me); vasoactive intestinal peptide; intestinal microbiota
8	14	2012	Metabolomics; systemic review; cell danger response; me; covid-19
9	13	2005	Treatment outcome; symptoms; people; validity; fatigue syndrome

CFS = chronic fatigue syndrome.

## 4. Discussion

Our investigation unveils worldwide patterns in the literature that receives the most citations within the domain of CFS research. Through the utilization of robust metrics, bibliometric analysis enables the evaluation of published papers, furnishing medical researchers in this field with vital insights regarding distinctive research areas and prospective avenues.

### 4.1. General information of the included papers

It is worth mentioning that the literature included in our study exhibited a wide range of time spans, with the most recent publication in 2021. This particular study employed systematic review and comparative methods to explore the clinical manifestations and symptomatology of long-term COVID and CFS, highlighting the growing focus on investigating the association between these 2 conditions and providing a platform for further research.^[17]^

Furthermore, your observation regarding the citation frequency reveals that the first 20 papers accounted for approximately 41% of the total citations, thus reinforcing the credibility of the conclusions drawn.

Through our analysis of the authors in the literature, we identified a substantial number of researchers dedicated to CFS studies, often engaging in multidisciplinary collaboration. Notably, influential authors such as Weinshenker, BG and Wingerchuk, DM emerged, indicating that their work holds significant potential as a research hotspot.

Additionally, we found that the article on international consensus standards by Carruthers, B.M., published in 2011, garnered the highest total citation frequency and average annual citation.^[1]^ This underscores the fundamental impact of this study on the field of CFS.

In terms of journals, Vrije Univ Brussel emerged as the most prolific publisher and garnered the highest citation frequency. Furthermore, journals such as Univ Bergen, Riga Stradins Univ, and HAFkeland Hosp have surfaced as emerging platforms for CFS research.

Among the countries contributing to CFS literature, the United States took the lead with the publication of the most papers (N = 37) and notable contributions to the diagnostic criteria and pathogenesis of CFS. This was followed by the United Kingdom (N = 32) and Belgium (N = 27). The combined articles from these countries accounted for more than half of the total literature, emphasizing their significant role in driving CFS research.

### 4.2. Future perspective

CFS is a complex disorder with disputed global prevalence. Epidemiological investigations have been conducted by various countries and organizations; however, due to inconsistent diagnosis and reporting, as well as differences in understanding CFS, accurately determining the global incidence rate remains challenging.^[13,18,19]^ In 2020, Lim, Eun-Jin published a review and meta-analysis analyzing studies conducted between 1990 and 2019, encompassing data from different regions and populations. The review presents an overview of CFS prevalence studies, but does not provide an exact global prevalence rate (adults: 0.1–2.8%; children or adolescents: 0.1–3.2%).^[19]^ Differences in prevalence may be attributed to variations in research methods, diagnostic criteria, and study populations, necessitating the collaboration of future scholars in establishing unified standards.

Various treatment approaches exist for CFS, and comprehensive treatment plans, including cognitive-behavioral therapy, psychological support, and moderate exercise, have been incorporated as evidence-based treatment options in the literature. Additionally, we observed that high-quality randomized controlled trials implemented placebo groups as a comparative baseline, suggesting the importance of incorporating placebo groups in future randomized controlled experiments to determine the presence of true treatment effects.^[9,20–22]^

Currently, researchers aim to discover more accurate and specific biomarkers through research and technological advancements to improve the diagnosis, monitoring, and treatment of CFS. However, precise biomarkers that serve as specific diagnostic indicators for CFS have not yet been identified. Therefore, in future studies, particularly in immunology and metabolomics, researchers will continue to focus on investigating biomarkers of CFS.^[23–26]^

## 5. Limitations

1. Although WOS is a widely used literature search database, it cannot be ruled out that it may not cover all older publications.
2. To ensure accurate analysis, we adopted a title keyword search method rather than a subject keyword search strategy. Our search results are precise but may not be comprehensive enough.
3. It should be noted that literature closer to the deadline typically has fewer citations, which decreases the likelihood of being included in the analysis. Therefore, incorporating a year-on-year conversion functionality into bibliometric analysis software is of significant importance.

## 6. Conclusion

Based on our understanding, this is the first quantitative assessment of the most frequently cited literature on CFS. Our research findings indicate that many journals have reported studies on CFS, particularly in the fields of medicine, clinical practice, and healthcare.

Future research directions in this field include the search for biomarkers, with a specific focus on immunology; updating diagnostic techniques; screening for risk genes associated with CFS; and conducting epidemiological investigations, among others.

## 7. Recommendations

*Establishing diagnostic criteria*: The current diagnostic criteria for CFS remain controversial. It is recommended to further research and develop standardized diagnostic criteria to enhance the accuracy and consistency of CFS diagnosis.

*Investigating etiology and pathogenesis*: Limited understanding exists regarding the etiology and pathogenesis of CFS. It is suggested to intensify research efforts in the etiology of CFS, including genetic, environmental, and immunological factors, in order to better comprehend and treat this condition.

*Exploring effective treatment approaches*: Current treatment options for CFS offer limited efficacy. It is advised to conduct additional clinical trials and research to explore novel treatment methods and medications, with the goal of improving patients’ quality of life and degree of recovery.

*Enhancing patient management and support*: The management and support of CFS require a multidisciplinary approach. It is recommended to strengthen collaboration among clinical physicians, psychologists, rehabilitation therapists, and social workers to provide comprehensive support and management for patients, encompassing medical, psychological, and social aspects.

*Improving public awareness and education*: CFS is a relatively unfamiliar condition. It is suggested to increase public awareness and education about CFS, in order to reduce discrimination and misconceptions towards CFS patients and raise societal attention to this illness.

*Supporting scientific research*: Research on CFS still faces challenges, including limitations in research funding and sample acquisition. It is proposed that governments and relevant organizations increase financial support for CFS research and provide additional sample resources to propel the progress of CFS research.

These recommendations aim to promote further development in understanding, prevention, diagnosis, and treatment of CFS, with the hope of providing some reference and guidance for clinical practitioners and researchers.

## Acknowledgments

The authors thank the softwares of Microsoft Excel, CiteSpace, v. 6.2. R4, andVOSviewer version 1.6.18, Scimago Graphica 1.0.35, and the Online Analysis Platform of Literature Metrology (http://bibliometric.com/).

## Author contributions

**Conceptualization:** Jun Chen.

**Data curation:** Xingxin Wang, Xuhao Li, Tiantian Dong, Wenyan Yu.

**Funding acquisition:** Jun Chen.

**Project administration:** Jun Chen.

**Software:** Zhixia Jia.

**Writing – original draft:** Xingxin Wang, Xuhao Li.

**Writing – review & editing:** Jun Chen.
